# Expression and Relations of Unique miRNAs Investigated in Metabolic Bariatric Surgery: A Systematic Review

**DOI:** 10.1007/s11695-024-07302-5

**Published:** 2024-06-25

**Authors:** Mohamed Hany, Hala M Demerdash, Anwar Ashraf Abouelnasr, Bart Torensma

**Affiliations:** 1 Department of Surgery, Medical Research Institute, Alexandria University, Alexandria, Egypt; 2 Madina Women’s Hospital (IFSO certified center, European chapter), Alexandria, Egypt; 3 Consultant and Professor of Clinical Pathology, Alexandria University, Alexandria, Egypt; 4 Leiden University Medical Center (LUMC), Leiden, The Netherlands

**Keywords:** miRNA, Review, Bariatric surgery, Weight loss, Associated medical problems

## Abstract

Several studies have indicated that miRNAs play crucial roles in adipogenesis, insulin resistance, and inflammatory pathways associated with obesity and change after metabolic bariatric surgery (MBS). This systematic review explores and maps the existing literature on how miRNAs are expressed and investigates the unique miRNAs with the effects after MBS. The Cochrane Central Register of Controlled Trials (CENTRAL), PubMed, and EMBASE were searched from 2019 until February 2024. This SR found 825 miRNAs from 25 studies, identifying 507 unique ones not used twice in the same study. A total of 21 studies (84%) measured RNA before and after surgery. The miRNA used per study ranged from 1 to 146 miRNA types, with a median study sample size of just 27 patients per study, raising concerns about some conclusions’ robustness. From the 507 unique miRNAs, only 16 were consistently analyzed in 4 to 7 studies, which gave 77 different outcomes in relation to miRNA after MBS. MiRNA 122 and 122-5p were analyzed the most. Others were 106b-5p, 140-5p, 183-5p, 199b-5p, 20b-5p, 424-5p, 486-5p, 7-5p, 92a, 93-5p, 194-5p, 21-5p, 221, 320a, and 223-3p. A gap was observed in many studies, whereby the results were not the same, or there was no explanation for the effects after MBS was given within the same miRNA. Fifteen miRNAs were reported to have the same upward and downward trend, although not within the same study, and only 26.1% employed some form of statistical modeling to account for bias or confounding factors. Directions and effects in miRNA are visible, but still, inconsistent outcomes linked to the same miRNA after MBS, underscoring the need for clarity in miRNA-outcome relationships. Collaborative efforts, consensus-driven miRNA dictionaries, and larger, more rigorous studies are necessary to improve methodology designs and improve outcomes.

**Supplementary Information:**

The online version contains supplementary material available at 10.1007/s11695-024-07302-5.

## Introduction

The intricate interplay between genetics and metabolic regulation has always been at the forefront of research in patients with obesity. As early as the 1990s and 2000s, the role of genetics in obesity was being elucidated with the discovery of genes like the leptin gene [1]. However, it was not until the advent of high-throughput RNA sequencing techniques in the twenty-first century that the research community began to unravel the complexities of non-coding RNAs, especially microRNAs (miRNAs), in the context of obesity and metabolic disorders [2]. microRNAs (miRNAs) are a class of small, non-coding RNA molecules, typically comprising about 18–25 nucleotides in length. These molecules play a pivotal role in post-transcriptional regulation of gene expression. miRNAs exert their function primarily by binding to the 3′ untranslated region (3′ UTR) of target messenger RNA (mRNA) molecules, leading to mRNA degradation or translational repression. As a result, miRNAs are instrumental in regulating various cellular processes, including differentiation, proliferation, apoptosis, and metabolism. Due to their extensive regulatory functions, miRNAs are implicated in multiple physiological and pathological processes, including those associated with obesity and metabolic disorders [3]. Several studies have since indicated that miRNAs play crucial roles in adipogenesis, insulin resistance, and inflammatory pathways associated with obesity [4]. Langi stated that a similar trend for miRNA was identified in 93-5p, 106b-5p, 7b-5p, 7i-5p, 16-5p, 19b-3p, 92a-3p, 222-3p, 142-3p, 140-5p, 155-5p, and 320-3p, which were reported to have lower expression levels. At the same time, 7-5p and 320c had increased expression levels after MBS, but it was unclear how this effect was related to the procedure or the effect on the patient itself [5]. The interest in metabolic bariatric surgery (MBS) was piqued when post-surgical metabolic improvements were attributed to weight loss and complex molecular and genetic modifications, including alterations in miRNA profiles [6]. While numerous individual studies have explored the role of specific miRNAs in MBS outcomes, a consolidated overview still needs to be provided. Given the field’s rapid evolution, collating existing data, understanding the breadth and depth of current research, and identifying potential overlaps or discrepancies in the findings is essential.

## Methods

This systematic review (SR) was conducted according to the PRISMA Reporting Items for Systematic Reviews and Meta-Analyses (PRISMA) guidelines by Moher et al. [7]. (Checklist appendix 1). All relevant and present RNA and MBS studies were collected for this SR and were registered at Prospero CRD42023469596.

### Study Aim and Inclusion Criteria

This study aims to systematically explore and map the existing literature on miRNA expression in metabolic and bariatric surgery (MBS), with the specific objectives to:

1. Determine the unique miRNAs investigated in MBS.
2. Identify the most frequently studied miRNAs in MBS and assess their potential significance and relevance for future research.

#### Inclusion Criteria

The review included studies involving patients aged 18 years or older with a BMI of 40 kg/m^2^ or a BMI of 35 kg/m^2^ with associated medical problems (e.g., diabetes, hypertension). Studies were considered if they tested or described the effects of miRNA in the context of MBS.

#### Study Selection

The initial systematic review articles from Langi et al. [5] until 2019 was used as a fundament for all studies before 2019 and thoroughly extracted. The Cochrane Central Register of Controlled Trials (CENTRAL), PubMed, and EMBASE were searched from 2019 until February 2024. We used the following terms and their synonyms, which were truncated where necessary: (*miRNA AND Bariatric surgery) OR (microRNA AND Bariatric surgery) OR (messenger RNA AND bariatric surgery) *(Appendix 2).

Grey literature was also searched with a reference crosscheck to detect eligible articles not identified in the previous searches or SR. This search was conducted without restrictions on the language.

## Type of Studies

### Included

Randomized controlled trials (RCTs), prospective and retrospective cohort studies, cross-sectional studies (CS), and case-control studies were included.

### Excluded

Descriptive studies, case series, and case reports were excluded because of their reduced level of evidence.

## Data Extraction

Two reviewers (BT and MH) independently screened the titles and abstracts based on the inclusion criteria, miRNA and MBS. After that, the same reviewers independently reviewed the remaining full-text reports for eligibility.

## Assessment of Risk of Bias

Two reviewers (BT, MH) independently assessed the risk of bias for the methodological quality of each included study using the Newcastle-Ottawa quality assessment scale for cohort studies, which is divided into three domains (selection bias, comparability, and outcome bias), what includes eight questions. The maximum score is nine points. High quality: 7–9 points, moderate quality: 4–6 stars, and low quality: 0–3 stars [8].

## Outcomes per miRNA

To understand the number of miRNAs cited in the literature, we performed a snapshot and chose to examine the most presented miRNA in four or more studies. This approach allowed us to survey the landscape of miRNAs in published research, understand the rationale or associations behind their selection, and discern patterns in analyzing specific miRNAs.

## Statistical Analysis

All the studies were extracted from Jotform Inc. (4 Embarcadero Center, Suite 780, San Francisco, CA 94111) and analyzed in R studio with R markdown, data.table, knitr, stringr,skimr, and ggplot2 (Version 2023.06.0+421). Categorical variables were expressed as n (%). Continuous normally distributed variables were expressed with their means and standard deviations, while non-normally distributed variables were expressed with their medians and min-max ranges. An algorithm was created to test for unique miRNA sets and effects in the miRNA presented in the studies.

## Results

### Search Strategy

**Fig. 1:**
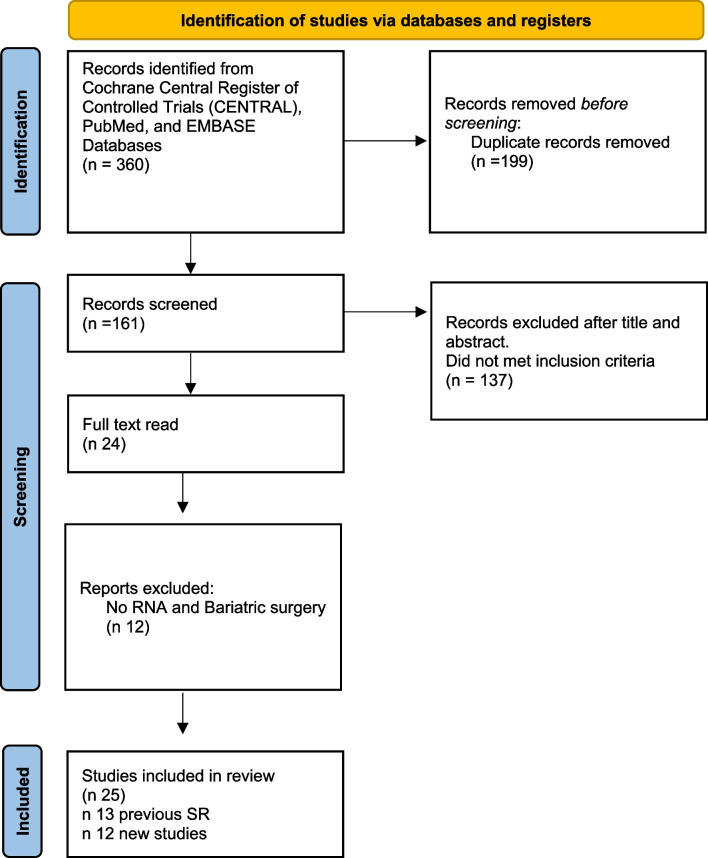
Identification of studies via databases and registers

A total of 360 studies were identified. After removing duplicates, 161 studies were screened on title and abstract. After evaluating 24 full-text articles, this systematic review included 9 new studies from our search that met our inclusion criteria; furthermore, when performing a grey literature search, three studies were found. 12 new studies and 13 previous from the SR were included (a total of 25) [4, 9–32] (PRISMA Flowchart Fig. 1).

### Baseline Characteristics

Upon aggregating data from all the included studies, the median number of patients per study was 27, ranging from 5 to 202. The median age was 45.8 years ±8.75 years. The median Body Mass Index (BMI) was 49.0 kg/m^2^ ±10.0, and postoperative BMI 29.6 kg/m^2^ ±3.4. Females constituted a median of 82% (min-max30%-100%). Regarding MBS procedures, 11 studies (44.0%) employed Roux-en-Y Gastric Bypass (RYGB), 3 (13.0%) utilized Laparoscopic Sleeve Gastrectomy (LSG), 2 (8.7%) opted for Laparoscopic Adjustable Gastric Banding (LAGB), 4 (17.4%) used either RYGB or LSG, and in 5 studies (20.0%), the procedures were either not specified or not mentioned. 21 (84%) of the RNA was measured before and after surgery, 1(4.0%) was performed cross-sectional between groups with unknown procedures, 1(4.0%) during surgery, and 2(8.0%) after MBS surgery was only measured. A detailed summary of each study’s overview, hypothesis, and outcomes can be found in Table 1.

**Table 1:** Studies information

Study	Total patients	Age (y)	Pre BMI (kg.m^2^)	Post BMI	Female	Type of BMS	RNA measured	Hypothesis	Outcome
Yeh 2022	*N*=150	36.0±10.0	40.9±5.5	Not mentioned	*N*=90 (60.0%)	Not mentioned	Before and after surgery	Expressed miRNAs in the serum of patients with an effective response after BMS compared with those without.	Panels of the serum ratios of miR-328-3p/miR-31-5p or miR-181a-5p/ miR-31-5p and individual BMI value exhibited good performance in preoperative prediction of treatment effectiveness
Hany 2022	*N*=55	34.2±11.3	44.9±6.8	31.9±4.7	*N*=36 (65.5%)	LSG	Before and after surgery	Evaluated the levels of two circulating microRNAs (miRNA-222 and miRNA-146a) before and after LSG and the effect on weight loss	LSG-mediated weight loss affected the plasma levels of miR146a and miR222-5p.
Eikelis 2021	*N*=19	39.2±8.1	42.1±4.5	Not mentioned weight stable (±3%) in the previous 6 months	*N*=14 (73.7%)	LAGB	During surgery	Total RNA was extracted from the liver and matched subcutaneous and visceral fat.	Reduction of miR132 may be a target for the regulation of liver lipid homeostasis and control of obesity-related blood pressure.
Breininger 2021	*N*=44 *N*=24 patients with obesity *N*=20 control	47.0±1.2	41.7 ±1.4	31.8 ±1.1	Patients with obesity: *N*=18 (82.0%) Control: *N*=12 (60.0%)	RYGB	Before and after surgery	Quantified 112 microRNA expression in rectal mucosal biopsies to investigate the likely functional consequences of these epigenetic changes	Weight loss, following RYGB, reduced expression of miR-31 and miR-215 to levels comparable with controls.
Sangiao-Alvarellos 2020	*N*=202 *N*=155 patients with obesity *N*=47 control	45.3±9.2	Patients with obesity 49.2±8.54 Control 39.7±10.9	After 12 months 30.6±4.8	Patients with obesity *N*=108 (69.7%) Control *N*=27 (57.4%)	RYGB or LSG	Before and after surgery	30 miRNAs in 155 patients with severe obesity and 47 controls and defined associations between miRNAs and metabolic parameters.	Higher circulating levels of liver-related miRNAs, such as miR-122, miR-885-5 p, or miR-192 were observed in morbidly patients with obesity patients.
Macartney-Coxson 2020	*N*=15	44.0±10.0	47.6±11.3	29.3±5.5	15 (100.0%)	RYGB	Before and after surgery	That miRNA expression levels would differ in adipose tissues before and after RYGB and associated weight-loss.	One omental, and thirteen subcutaneous adipose miRNAs were significantly, differentially expressed after gastric bypass, including down-regulation of miR-223–3p and its antisense relative, miR-223–5p, in both adipose tissues
Doyon 2020	*N*=20 Low weight loss *N*=10 High weight loss *N*=10	53.5 (24-67)	Low: 47.1 (40.8–50.8) High: 39.7 (36.6–48.7)	After 12 months Low: 35.2 (32.3–41.5) High: 26.5 (23.9–28.5)	*N*=15 (75.0%)	RYGB or LSG	After surgery	To evaluate the feasibility of using miRNA to predict weight loss after bariatric surgery	In total, of the 119 miRNA detected in the serum of the patients, 7 demonstrated potential for discriminating between the high and low weight loss groups.
Cereijo 2020	*N*=26	48.92 (20–64)	45.66 (35–65)	After 6 months 33.11 (23-51)	*N*=16 (61.5%)	RYGB or LSG	Before and after surgery	Evaluated circulating levels of miR-92a and miR99b, two miRNAs proposed as biomarkers of brown fat activity	Serum miR-92a levels were strongly reduced at 6 months after BS, reaching levels similar to those in controls. miR-99b levels were unchanged
Bae 2019	Patients with obesity *N*=16 Healthy volunteers *N*=18	Patients with obesity 31.3 ± 8.76 HV 38.6 ± 7.9	Patients with obesity 39.9 ± 4.6 HV 21.6 ± 1.4	31.00 ± 4.68	Patients with obesity *N*= 9 (56.3%) HV 13 (72.2%)	RYGB or LSG	Before and after surgery	Serum exosomal miRNA in patients 6 months after surgery.	Patients with obesity have a distinct exosomal miRNA expression profile compared with HVs. In addition, weight loss after surgery alters the exosomal miRNA profile of patients with obesity
Atkin 2019	*N*=29	47.5 ± 8.5	46.2 ± 9.9	42.4 ± 9.3	*N*= 20 (69.0%)	RYGB	Before and after surgery	This study investigated 175 miRNA whether or not patients with pre-surgical type 2 diabetes had changes in microRNAs immediately following bariatric surgery.	Changes from pre- to post-surgery in 32 of 175 microRNAs were nominally significant (*p* < 0.05). Seven microRNAs showed significant changes 21 days after bariatric surgery. Functional pathways of the altered microRNAs were associated with diabetes-, pituitary-, and liver-related disease, with expression in natural killer cells, and pivotal intestinal pathology suggesting possible mechanistic roles in early diabetes responses following bariatric surgery.
Wang 2018	*N*=124	46 (25-66)	31.88 ± 2.76	26.31 ± 3.82	*N*= 78 (62.9%)	Unknown	Before and after surgery	Investigate the value of peripheral blood miR-448 and its target gene SIRT1 in predicting the treatment efficacy of laparoscopic bariatric surgery in patients with obesity T2DM patients.	The results demonstrated that miR-448 and its target gene SIRT1 can serve as prognostic indicators for patients with obesity T2DM patients after laparoscopic bariatric surgery
Chien-Hung Liao 2018	Patients with obesity *N*=20 Control *N*=8	Patients with obesity: 34.1 (9.9) Control 49.5 (17.7)	Patients with obesity: 42.4 (6·6) Control 24.6 (3.8)	-	Patients with obesity: *N*= 13 (65%) Control *N*=4 (50%)	LSG	After surgery	The present study aimed to compare the miRNA signatures between VF and SF and study their influences on outcomes of bariatric surgery	Higher miR-122 in VF may be associated with greater body weight loss after bariatric surgery.
Hohensinner 2018	*N*=58	41.9 ± 11.2	43.98 ± 3.55	28.02 ± 4.08	*N*= 41 (70.7%)	RYGB	Before and after surgery	To evaluate markers of premature aging including SASP and miRNAs, telomere length, and telomere stability in patients before and 2 years after gastric bypass surgery	The aging-associated miRNA miR10a_5p was upregulated significantly (*p* = 0.039), while the other tested miRNAs showed no difference
Alkandari 2018	*N*=9	46.1 (9.8)	49 (10)	30.7 (4.5)	*N*= 5 (55.6%)	RYGB	Before and after surgery	To assess the temporal effect of bariatric surgery on 179 validated circulating microRNA expression profiles.	RYGB altered the circulating microRNAome in a time dependent manner and the expression of 48 circulating microRNAs were significantly different.
Yo Nunez Lopez 2017	Exercise *N*=11 Control *N*=11	Exercise 43.0 (36.7–49.3) Control 38.5 (30.3–46.6)	Exercise 39.5 (36.1–42.8) Control 40.8 (36.8–44.7)	-	*N*= 9 (81.8%)	RYGB + exercise and control groups	Before and after surgery	Evaluated the changes in plasma levels of 94 metabolically involved miRNAs in morbidly patients with obesity subjects that underwent RYGB surgery-induced weight loss with or without an exercise program	In the control group, weight loss significantly altered the pattern of circulating miR-7, miR-15a, miR-34a, miR-106a, miR-122, and miR-221. In the Exercise group, a distinct miRNA signature was altered: miR-15a, miR-34a, miR-122, miR-135b, miR-144, miR-149, and miR-206
Nardelli 2017	Patients with obesity *N*=3 Control *N*=2	Patients with obesity 48 years Control 37 years	Patients with obesity 42.9 Control 21.5	32.0	*N*=5 (100.0%)	LAGB	Before and after surgery	The aim of the present study was to investigate the SAT miRNA profile in three women with severe obesity before (T0) and 3 years (T1) after LAGB and to evaluate whether miRNAs are involved in post-LAGB metabolic improvement	miRNA profile changes after LAGB. Decreased levels of miR-212, miR-299-5p, and miR-671-3p after 3 years concur in reducing SAT inflammation.
Mysore 2017	*N*=22	48±10	43.4±5.0	29.4±5.7	*N*=22 (100.0%)	RYGB	Before and after surgery	Understanding the relationship of adipose tissue and adipocyte ANGPTL8 expression with hallmark insulin-sensitive genes ADIPOQ and SLC2A4/GLUT4 (23, 24), inflammation, and miRNAs associated with obesity and T2D	miRNA-221-3p impact may become especially prominent under pathologic conditions such as morbid obesity, putatively contributing to the impaired AT lipid metabolism in metabolic disease
Kuryłowicz 2017	Patients with obesity *N*=58 Normal weight control group *N*=55	Not mentioned	>40 kg/m2	-	Not mentioned	Unknown	Before and after surgery	NGS for the identification of miRNAs, the expression of which varies between different adipose tissue depots of patients with obesity before and after weight loss, as well as between obesity and normal-weight patients	NGS identified 344 miRNAs expressed in adipose tissues with ≥5 reads per million. miRNA profiles are possibly associated with changes of weight responsible for a differential regulation of molecular pathways in adipose tissue when the individual has obesity, and after the individual has lost weight.
Hubal 2017	*N*=6	38.5±6.8	51.2±8.8	32.6±8.1	*N*=6 (100.0%)	RYGB	Before and after surgery	Biological pathways analysis to predicted mRNA targets of 168 surgery-responsive miRNA’s identifying the insulin signaling pathway	Insulin signaling pathway were target in 10 miRNAs correlated to changes in HOMA
Blum 2017	*N*=10	Unknown	Unknown	Unknown	*N*=3 (30.0%)	LSG	Before and after surgery	Hypothesized that levels of specific circulating miRNAs are altered following surgery and may contribute to lower cancer risk. Approximately 50 miRNAs could be identified	The serum miR-122/miR-451 ratio may serve as a marker for endothelial function in patients with obesity. miR122 is the dominant miRNA in the liver and a known tumor suppressor.
Ortega 2015 Inflammation	Unknown	Unknown	Unknown	Unknown	Unknown	The miRNAs that experienced the most dramatic changes were studied in subcutaneous human adipose tissue before and approximately 2 years after bariatric surgery-induced weight loss.	Before and after surgery	The study aimed to characterize the changes induced by inflammation on the miRNA pattern of human adipocytes and macrophages on an extensive profile of 754 common miRNAs was assessed in cells (human primary mature adipocytes, and the macrophage-like cell line THP-1) and in their supernatants (SN) using TaqMan low-density array	Inflammation induces a specific miRNA pattern in adipocytes and M1 macrophages, with impact on the physiopathology of obesity-induced inflammation of adipose tissue
Ortega 2015 (surgery)	*N*=25	48±10	43.1±4.9	29.2±5.5	*N*=25 (100.0%)	RYGB	Before and after surgery	To identify changes in messenger RNA (mRNA) and miRNA expressions and their interaction in human AT before and after surgery-induced weight loss	A total 15 miRNAs were differentially expressed after surgery-induced weight loss. Findings suggest impaired miRNA target gene expression in patients with obesity AT in close association with inflammation, both improving after weight loss.
Lirun 2015	*N*=15	27.7±2.4	35.4±1.5	28.9±0.9	*N*=9 (60.0%)	RYGB	Before and after surgery	This study setup was to find some certain microRNA that changed greatly after RYGB with BMI decrease	After RYGB, there was an obvious change in the serum microRNA expression of both low- and high-BMI groups compared with those before operation. The expression of let-7, miR-24, miR-24-23a/b, miR-24-93, miR-24-26a, miR-24-151-3p, miR-24-425, miR-24-151-5p, miR-24-146a, and miR-24-103a were downregulated, whereas miR-4787-5p and miR-24-1281 were upregulated.
Ortega 2013	Patients with severe obesity *N*=8 Obesity *N*=12 Control *N*=12	Severe obesity 46±5 Obesity 51±8 Control 50±11	Severe obesity 45.3±6.3 Obesity 33.1±6.5 Control 23.7±1.1	28.9±4.3	Unknown	Unknown	Cross-sectional between groups	The aim of this study was to describe the circulating miRNA profile for humans according to specific degree of obesity. The effects of surgery and diet-induced weight loss on circulating miRNAs were also investigated in independent cohorts.	Patients with severe obesity showed a marked increase of miR-140-5p, miR142-3p (and miR-222) and decreased levels of miR-532–5p, miR-125b, miR130b, miR-221, miR-15a, miR-423-5p, and miR520c-3p Circulating miRNAs are deregulated in severe obesity. Weight loss–induced changes in this profile and the study of in silico targets support this observation and suggest a potential mechanistic relevance
Hulsmans 2012	Patients with severe obesity *N*=21 Control *N*=14	Patients with severe obesity 39±3.0 Control 33±3.0	Patients with severe obesity 44±1.0 Control 21±1.0	36±1.0	Patients with severe obesity *N*=16 (77.0%) Control *N*=10 (73.0%)	RYGB	Before and after surgery	Identify miR families dysregulated in monocytes of patients with obesity patients with putative targets in the TLR/NFkB signaling pathway and with restored expression profile after weight loss	A total of 133 miR were differentially expressed. miR-181a, -181b, and -181d, identified as possible regulators of the TLR/NFkB signaling, were decreased in patients with obesity monocytes, and weight loss normalized their expression

*miRNAs* microRNA, *BMS* bariatric metabolic surgery, *LSG* laparoscopic sleeve gastrectomy, *RYGB* Roux en Y gastric bypass, *LAGB* laparoscopic adjustable gastric banding, *TLR* toll-like receptor, *NFkB* nuclear factor kappa B, *BMI* body mass index, *AT* adipose tissue, *VF* visceral fat, *SF* subcutaneous fat, *NGS* next-generation sequencing

### miRNA

**Fig. 2:**
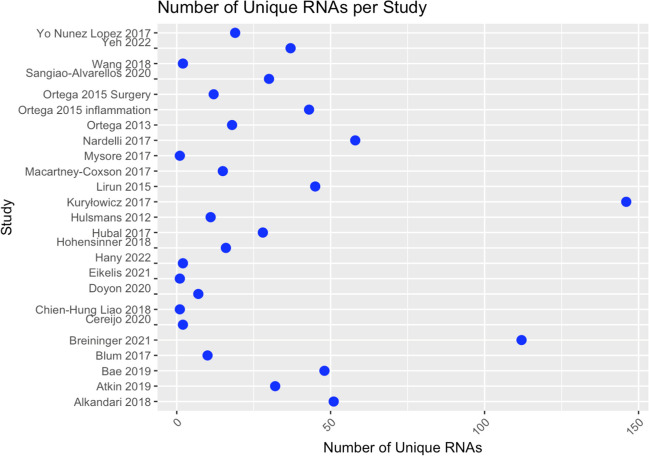
Number of Unique miRNA per study

From the studies we reviewed, we identified a total of 825 miRNAs. Upon further examination for unique miRNA counts, 507 distinct miRNAs (100%) were detected. The unique miRNAs tested in individual studies ranged from 1 to 146 (Fig. 2).

### Number of Studies with the Same miRNA

**Fig. 3:**
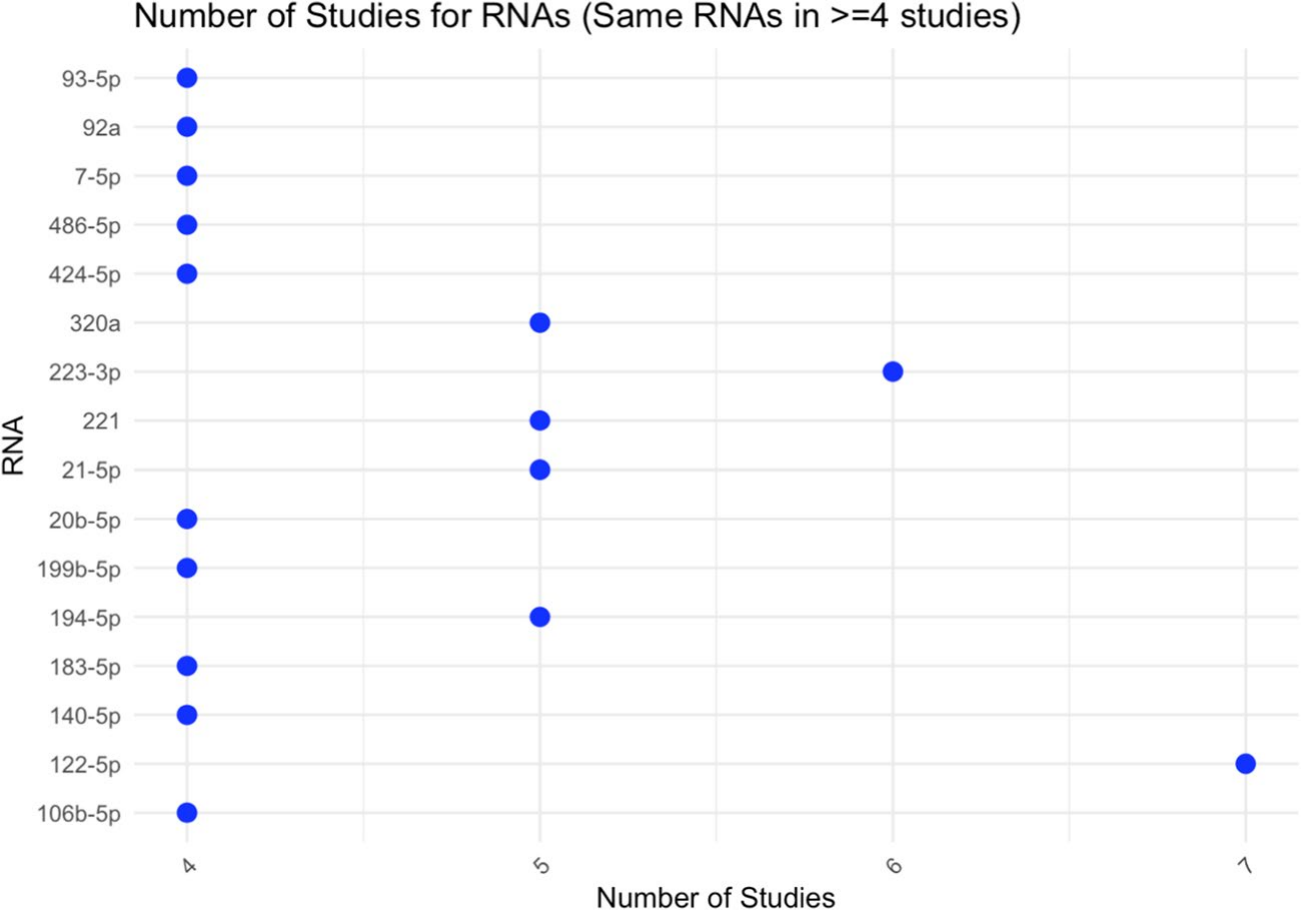
Number of studies with the same miRNA

Of the miRNAs identified in our review, 352 (69.4%) unique miRNAs were presented in single studies, 95 (18.7%) appeared in two studies, and 44 (8.7%) were presented in three studies. Four studies mentioned a smaller subset comprising 10 miRNAs (1.9%), including miRNA 106b-5p, 140-5p, 183-5p, 199b-5p, 20b-5p, 424-5p, 486-5p, 7-5p, 92a, and 93-5p. Additionally, 4 miRNAs (194-5p, 21-5p, 221, and 320a) were each found in five studies, representing 0.79% of the total. In contrast, miRNA 223-3p was the only miRNA, at 0.19%, in six studies (Fig. 3), and miRNA 122-5p (0.19%) to appear in 7 seven studies. For a comprehensive list of all miRNAs across the studies, refer to Appendix 3.

### Outcomes per miRNA

All miRNAs in 3 or fewer studies would give 484 different outcomes. Therefore, we looked at all miRNA in four or more studies, which gave 77 different outcomes, to better understand the miRNA in MBS research and provide more power in the outcome using more studies.

### miRNA 122/122-5p

In our analysis, miRNA 122 and 122-5p were mentioned the most in the reviewed studies. Specifically, miRNA 122 was mentioned in three distinct studies [4, 19, 26], while miRNA-122-5p was referenced in seven [9, 13, 16, 17, 22, 25, 32]

Blum et al. delved into the effects of MBS on vascular endothelial cells (VAC). Their research was geared towards understanding the potential role of MBS in preventing cancer and protecting VAC [26]. Chien-Hung Liao et al. explored how weight loss (WL) impacts visceral and subcutaneous fat [19]. Ortega et al. focused on understanding the standalone effects of weight loss, excluding other potential influences [4].

Focus on miRNA 122-5p, both Atkin et al. and Bae et al. investigated the implications of weight loss on miRNA 122-5p [16, 17]. However, their findings did not elaborate on the specifics or underlying mechanisms tied to this microRNA. Hubal et al. established a connection between miRNA 122-5p and changes in HOMA, highlighting the involvement of more than three insulin signaling targets in this relationship [25]. Sangiao et al. linked miRNA 122-5p to various outcomes related to the liver [13].

Yeh et al. tested serum microRNA panels and found no significant effect on 122-5p in the algorithm to identify circulating miRNAs after MBS (most influenced effect were 877-5p, 1285-3p, 328-3p, 181a-5p, 3688-3p, 31-5p) [9]. Lastly, Yo Nunez et al. [22] did mention miRNA 122-5p in the study; they pointed out that it was not among the top three miRNAs that exhibited the most pronounced effects post-weight loss. Instead, Nunez identified the most influential miRNA 328-3p, 181a-5p, and 31-5p. Interestingly, 328-3p and 181a-5p miRNAs were only used in Nunez and Yeh et al. [9, 22] was not identified by other studies as the most pronounced, and 31-5p was in three studies, the most prominent [9, 12, 22]. Kuryłowicz et al. found an effect whereby 122-5p was involved in inflammation and immune response with no further explanation [32].

### miRNA 92A

Cereijo et al. found in glucose hemostasis a connection to elevated levels of 92A [15]. Lirun et al. observed that 92A was downregulated following RYGB, but they did not further investigate its association with other associated medical problems (AMP) [29]. Similarly, Nardelli et al. reported an upregulation of 92A in patients with obesity, but they did not extend their study to explore its relationship with other AMPs [23]. Ortega et al. identified a link between 92A and inflammation related to obesity, suggesting that inflammation could induce elevated 92A levels [27].

### miRNA 7-5p

Atkin et al. explored the significant effects following MBS and established links to type 2 diabetes (T2D), non-alcohol fatty liver disease (NAFLD), and liver fibrosis. Additionally, their research indicated connections to the release of adrenal and thyroid hormones [17]. Bae et al. observed increased levels post-operatively but did not extend their investigation to other AMP [16]. Although Breininger et al. observed some effects of miRNA following MBS, these did not make it to their final model or secure a spot among the top 5 miRNA effects. Specifically, they reported upregulation of 31-5p, 215-3p, 215-5p, 30a-5p, and 204-5p, and downregulation of 1273f, 200b-5p, 1247-5p, 552-5p, and 1247-3p. Additionally, they did not extend their research to other AMPs [12]. Nunez-Lopez et al. identified a relationship in WL after MBS, but they, too, abstained from exploring its associations with other AMPs [22].

### miRNA 221

Lirum et al. observed an effect presented, but they did not investigate further regarding other AMPs [29]. Ortega et al. identified decreased levels in cases of severe obesity, and after WL, they noticed an upregulation. They also observed increases within different BMI classes that change is linked to the fat mass [4]. Another study by Ortega et al. found a connection to inflammation in obesity [27]. Furthermore, a third study from Ortega et al. reported that miRNA 221 levels decreased post-surgery. The only significant effect was also observed with miRNA 155, where 2 out of the 12 tested miRNAs showed this change [28]. Finally, Nardelli et al. found differences in miRNA 221 levels between patients with and without obesity but did not extend their research to other AMPs [23].

### miRNA 223-3p

Atkin et al. observed a significant downregulated effect following MBS, but they did not investigate further regarding other AMPs [17]. Breininger et al. identified miRNA 223-3p, although they did not specify which effects [12]. Hohensinner et al. noted an effect after RYGB on this miRNA but did not detail them due to their lack of significance [20]. Similarly, Sangiao-Alvarellos et al. detected some effects after MBS but no results associations with other AMPs [13]. Kuryłowicz et al. found involvement in inflammation and immune response and down-regulated after MBS [32]. Macartney-Coxson et al. found in one omental and thirteen subcutaneous adipose tissues that miRNA 223–3p was significantly differentially expressed with down-regulation after RYGB [31].

### miRNA 302A

Alkandari et al. observed an effect following MBS combined with WL and linked this effect to fasting glucose levels [21]. Blum et al. indicated a change post-MBS but did not provide further details in the result section [26]. Breininger et al. detected some effects in the results of 302-A. Still, they did not specify the nature of these effects [12]. Hohensinner et al. acknowledged its presence in their initial findings, but its overall impact was insignificant [20]. Lirun et al. noted that miRNA 320A was upregulated and exhibited the most pronounced effect in individuals with low BMI and linked an impact on T2D following MBS to this specific miRNA [29].

### miRNA 21-5p

Alkandari et al. identified a relationship between preoperative measures and those taken at 9 and 12 months post-operatively [21]. Atkin et al. observed a significant effect after MBS but did not elaborate on its specifics [17]. Hohensinner et al. found no significant change after MBS and refrained from providing additional explanations [20]. Sangiao-Alvarellos et al. detected some effects after MBS but no insights on how and what this effect was [13]. Kuryłowicz et al. found 21-5p was pro-adipogenic and upregulated in patients with obesity in subcutaneous adipose (SAT) tissue but no effect after MBS [32].

### miRNA 194-5p

Alkandari et al. identified a relationship between preoperative measures and those taken at 3, 9, and 12 months post-operatively. However, they did not find a significant effect at the 6-month mark [21]. Atkin et al. observed a significant effect after MBS but did not elaborate on its specifics [17]. Breininger et al. detected some results of the miRNA in question that did not specify their nature, [12]. Yeh et al. included miRNA 194-5p in their analysis, but it remains if any effect after MBS was evident [9]. Kuryłowicz et al. found that 194-5p was pro-adipogenic and downregulated in SAT adipose tissue after MBS [32].

### miRNA 140-5p

Bae et al. observed a down-regulated effect following MBS but did not provide additional details or conduct further testing [16]. Breininger et al. detected some results of the miRNA in question, though they did not specify their nature [12]. Ortega et al. identified an effect following MBS and WL, with a noted decrease in the miRNA levels [4]. Sangiao-Alvarellos et al. detected some effects after MBS in the created models, but they, too, did not offer further insights on how and what this effect was [13].

### miRNA 106b-5p

Alkandari et al. identified a relationship between preoperative measures and those taken 3 and 12 months post-operatively. However, they did not observe any significant effects at the 6- and 9-month marks [21]. Hohensinner et al. noted miRNA 106b-5p presence in their models, but it was not statistically significant as a marker [20]. Yeh et al. incorporated miRNA 106b-5p in their models, but it remains unclear whether it exhibited any impact [9]. Nunez-Lopez et al. associated the miRNA with cardiometabolic risk factors but refrained from providing additional explanations [22].

### miRNA 93-5p

Alkandari et al. found that 93-5p levels were significantly decreased in the early months following MBS [21]. Atkin et al. observed a significant effect after MBS but did not elaborate on its specifics [17]. Yeh et al. incorporated it in their models, but it remains unclear whether it exhibited any impact [9]. Kuryłowicz et al. found involvement in inflammation and immune response and downregulated after MBS [32].

### miRNA 20b-5p

Alkandari et al. identified a relationship between preoperative measures and 12 months post-operatively with a down-regulated effect but no effect in the previous months [21]. Atkin et al. observed a significant effect after MBS but did not elaborate on its specifics [17]. Sangiao-Alvarellos et al. detected some effects after MBS in the created models, but they, too, did not offer further insights on how and what this effect was [13]. Kuryłowicz et al. found involvement in inflammation and immune response and downregulated after MBS [32].

### miRNA 424-5p

Alkandari et al. identified a relationship between preoperative measures and 9 months post-operatively with a down-regulated effect not on 12 months [21]. Atkin et al. observed a significant effect after MBS but did not elaborate on its specifics [17]. Bae et al. found upregulated in patients with obesity and downregulated after MBS for 20b-5p [16]. Kuryłowicz et al. found involvement in oncogenisis response and down-regulation after MBS [32].

### miRNA 199b-5p

Bae et al. found it downregulated after MBS [16]. Breininger et al. detected some results of the miRNA in question, though they did not specify their nature [12]. Nardelli et al. found differences in miRNA 221 levels between patients with and without obesity but did not extend their research to other AMP [23]. Kuryłowicz et al. found it was pro-adipogenic and upregulated in SAT after MBS [32].

### miRNA 486-5p

Breininger et al. detected some results of the miRNA in question, though they did not specify their nature [12]. Lirun et al. found that 486-5p was significantly upregulated, which only appeared in the high-BMI group [29]. Sangiao-Alvarellos et al. detected some effects after MBS in the created models but did not offer further insights on how and what this effect was [13]. Kuryłowicz et al. found it with an unknown function but downregulated in SAT after MBS [32].

### miRNA 183-5p

Bae et al. found it was upregulated in patients between obesity and healthy volunteers [16]. Yeh et al. incorporated it in their models, but it remains unclear whether it exhibited any impact [9]. Nunez-Lopez et al. associated the miRNA with cardiometabolic risk factors but refrained from providing additional explanations [22]. Kuryłowicz et al. found involvement in oncogenisis response and downregulation after MBS [32].

### miRNa and Postoperative Direction

Following MBS and the subsequent weight loss, the value of specific miRNAs could either decrease (“down” direction) or increase (“up” direction). From the studies that reported these directional changes, 135 unique miRNAs (26.6%) exhibited a downward trend, while 101 (19.9%) showed an upward trend postoperatively. Intriguingly, we identified 15 miRNAs (representing 2.9% of the unique counts) that were reported to have both upward and downward trends, although not within the same study. These miRNAs include 106b-5p, 125b-5p, 15a-5p, 424-5p, 532-3p, 21, 31-5p, 92a, 429, 122, 221, 122-5p, 223-3p, 1246, and 128. The rest of the studies did not mention any direction or could not extract from the results. A comprehensive list detailing the post-operative directional changes of all miRNAs can be found in Appendix 4.

### Bias and Confounding Correction on miRNA

Of the studies reviewed, 6 (26.1%) employed some form of statistical modeling to account for bias or confounding factors. These methodologies ranged from adjusting for baseline characteristics to using logistic regressions and multivariate analyses for postoperative profiles [10, 13, 14, 17, 21, 22]

### Risk of Bias if Included Studies

**Fig. 4:**
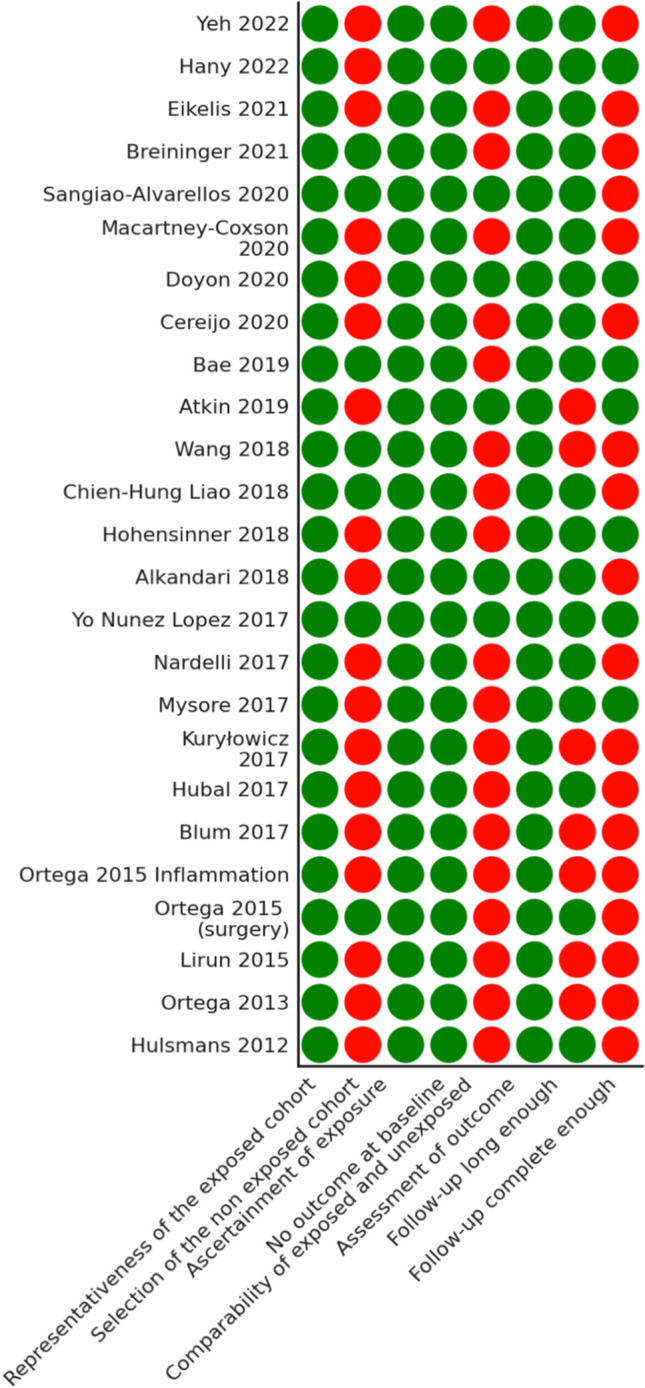
Risk of bias assessment

High quality was present in 6 out of 25 studies (24%) [10, 13, 16, 17, 21, 22]. Moderate quality was present in the rest of the 19 studies, ranging from 4 to 6; therefore, no low quality was present in the included studies (Table 2, Fig. 4).

**Table 2:** Risk of bias

	Selection				Comparability	Outcome			Total
	Representativeness of the exposed cohort (1 point)	Selection of the non exposed cohort (1 point)	Ascertainment of exposure (1 point)	No outcome at baseline (1 point)	Comparability of exposed and unexposed (2 points max)	Assessment of outcome (1 point)	Follow-up long enough (1 point)	Follow-up complete enough (1 point)	Max 9
Study									
Yeh 2022	+	−	+	+	-	+	+	-	5
Hany 2022	+	−	+	+	+	+	+	+	8
Eikelis 2021	+	−	+	+	−	+	+	−	5
Breininger 2021	+	+	+	+	−	+	+	−	6
Sangiao-Alvarellos 2020	+	+	+	+	+	+	+	−	8
Macartney-Coxson 2020	+	−	+	+	−	+	+	−	5
Doyon 2020	+	−	+	+	+	+	+	+	6
Cereijo 2020	+	−	+	+	−	+	+	−	5
Bae 2019	+	+	+	+	−	+	+	+	7
Atkin 2019	+	−	+	+	+	+	−	+	7
Wang 2018	+	+	+	+	−	+	−	−	5
Chien-Hung Liao 2018	+	+	+	+	−	+	+	−	6
Hohensinner 2018	+	−	+	+	−	+	+	+	6
Alkandari 2018	+	−	+	+	+	+	+	−	7
Yo Nunez Lopez 2017	+	+	+	+	+	+	+	+	8
Nardelli 2017	+	−	+	+	−	+	+	−	5
Mysore 2017	+	−	+	+	−	+	+	+	6
Kuryłowicz 2017	+	−	+	+	−	+	−	−	4
Hubal 2017	+	−	+	+	−	+	+	−	5
Blum 2017	+	−	+	+	−	+	−	−	4
Ortega 2015 Inflammation	+	−	+	+	−	+	−	−	4
Ortega 2015 (surgery)	+	+	+	+	−	+	+	−	6
Lirun 2015	+	−	+	+	−	+	−	−	4
Ortega 2013	+	−	+	+	−	+	−	−	4
Hulsmans 2012	+	−	+	+	−	+	+	−	5

## Discussion

In this systematic review, we identified 507 unique miRNA counts. Never have all miRNAs been investigated in detail before. Notably, only a fraction, 3.07%, of these miRNAs appeared in four or more studies. A total of 21 (84%) of the RNA was measured before and after surgery. Furthermore, a mere 2.9% exhibited mixed outcome directions, indicating that their levels could either increase or decrease following weight loss achieved post-MBS.

For several reasons, understanding the effects of miRNA changes post-MBS is crucial. miRNAs play a significant role in regulating gene expression and are involved in numerous metabolic processes, including glucose metabolism, lipid metabolism, and inflammation—areas directly impacted by MBS. Changes in miRNA expression following surgery could potentially reflect, influence, or predict the metabolic improvements observed in patients, such as weight loss, improved insulin sensitivity, and reduced risk of cardiovascular diseases. By elucidating these effects, miRNA profiling could serve as a valuable tool for predicting treatment outcomes, tailoring patient management, and even guiding the selection of surgical techniques based on individual miRNA profiles.

Previous reviews [5, 33] have either performed meta-analyses on specific subsets of miRNAs or delved into shifts in miRNA expression due to factors like lifestyle modifications; our review has cast a wider net. We have mapped the landscape of miRNA-MBS studies, emphasizing the diversity of miRNAs examined and the frequency of their investigation. We searched for relations between different MBS procedures and their effects on the patient. A previously well-performed SR by Langi et al. performed a meta-analysis to identify consistently modulated miRNAs after MBS and report biological pathways predicted to be regulated by miRNAs. The concluded inconsistencies spanned various aspects, including the source of the miRNA, the type of MBS employed, the duration of post-surgery observations, and the methodologies used for the miRNA profiling [5].

A distinct SR by Catanzaro et al. delved into contemporary research on shifts in miRNA expression among patients with obesity undergoing lifestyle modifications or MBS. Their review underscores burgeoning evidence that positions miRNAs as potential indicators of weight loss and their responsiveness to obesity intervention strategies, but it still could not identify all the aspects well [33].

### miRNA

We found that most presented miRNAs could not determine the specific miRNA effect on patients with obesity before or after MBS and what procedure had the best effect. The study design of the miRNA studies was highly variable. We found that miRNAs had both upward and downward trends, although they were not within the same study and did not follow the same procedure. This makes the interpretation of the clinical effect for patients with obesity after MBS very difficult.

Previously, Langi stated that a similar trend for miRNA was identified in 93-5p, 106b-5p, 7b-5p, 7i-5p, 16-5p, 19b-3p, 92a-3p, 222-3p, 142-3p, 140-5p, 155-5p, and 320-3p, which were reported to have lower expression levels. At the same time, 7-5p and 320c had increased expression levels after MBS, but it was unclear how this effect was related to the procedure or the effect on the patient itself, something we also concluded from our SR.

Our SR, compared to Langi et al., found that only the 140-5p, 93-5p, and 206-5p were found now in 4 studies (16%), and the same miRNA 16-5p, 19b-3p, 92a-3p were found in three studies (12%). The rest of the presented miRNA were presented in two or one study; therefore, they were not more reliable than the other 507 unique miRNA counts we found, all with a potential effect.

Furthermore, we identified 15 miRNAs that were reported to have both upward and downward trends, although not within the same study. These miRNAs include 106b-5p, 125b-5p, 15a-5p, 424-5p, 532-3p, 21, 31-5p, 92a, 429, 122, 221, 122-5p, 223-3p, 1246, and 128.

Langi reported in 2019 that 106b-5p and 92a had the same direction. Our new search identified the different directions, whereby 106-5b was measured in an RYGB and the other direction in an unknown procedure. For 92a, up and downward trends were seen in RYGB, LSG, LAGB, or unknown procedures. This also makes it very difficult to determine what miRNA has the best effect or if a procedure is reasonable for an upward or downward trend since this was impossible to test.

Also, a study by Alkandari et al. showed a different range of effects between specific time points. They tested 194-5p and found effects at 3, 9, and 12 months but not at 6 months. The same was true for 106-5p, which was significant at 3 and 12 months but not at 6 and 9 months. This study tested it in only nine patients so that the outcome can be seen as less reliable.

We also found that 352 miRNAs appeared once in one of the 25 studies. All have a possible effect but have not been validated in other studies, making them challenging to compare. In many instances, the same miRNA did not consistently correlate with the same outcome, and the relationship or nature of the association was often not delineated. This presents a clear challenge and underscores the need for more rigorous research to determine the best miRNA sets and their consistent associations.

In our review, miRNA 122 and 122-5p emerged as the most frequently used, appearing in ten studies. However, their effects post-MBS varied, with some studies reporting them as significant and others not. Also, no apparent effect was described for the type of procedure. It was measured in RYGB in four studies and twice in LSG, and the procedure was unknown in four studies, but it is unclear who had a more significant effect post-MBS. Furthermore, none of the 25 included studies tested the effect of various MBS procedures on a specific miRNA. Therefore, it is unclear if a particular type of procedure is superior to another. Some studies concluded that 328-3p, 181a-5p, and 31-5p had the most influential effect after MBS but were not the most cited miRNAs in our SR [9, 12, 22]. Intriguingly, these specific three miRNAs were also not presented as the best effect in two prior systematic reviews [5, 33] that posited other hypotheses.

This disparity in findings is further underscored by Yeh et al., who found that the most influenced effect was 877-5p, 1285-3p, 328-3p, 181a-5p, 3688-3p, 31-5p (overlap on 181a-5p and 31-5p) but all the rest of the miRNA was not present in other studies [9].

Breininger et al.’s [12] study highlighted several miRNAs in their final model. They ranked the top upregulated miRNAs as 31-5p, 215-3p, 215-5p, 30a-5p, and 204-5p, and identified the most downregulated as 1273f, 200b-5p, 1247-5p, 552-5p, and 1247-3p.

Notably, except 31-5p, none of these miRNAs were mentioned in either previous systematic review [5, 33], nor were they featured in other studies as potentially included in our review.

## Implications and Future Directions

Our systematic review reveals a significant gap in the current research landscape. The inconsistency in the directions of miRNA changes post-MBS and the limited number of studies with low power of included patients, in which any given miRNA has been consistently observed, pose a significant challenge for clinical implications and effects. Given the inconsistency across studies and the lack of consistent testing of the same miRNA over time, it was impossible to pool the data and identify specific patterns that were robust enough to draw clinical conclusions about the effects of an miRNA before and after miRNA, besides the fact an miRNA will go down or upwards after MBS. Only a tiny fraction of identified miRNAs appeared in four or more studies, and an even smaller percentage exhibited consistent trends. This inconsistency complicates the ability to draw definitive conclusions about these miRNAs’ roles in the metabolic changes observed after MBS.

For future research, this highlights the need for a more standardized approach to studying miRNAs associated with MBS. A pressing requirement is for larger, more rigorous studies to provide clearer insights into how miRNA profiles change in response to different surgical procedures. Research should aim to standardize the methodologies for miRNA measurement and enhance the comparability of studies to ensure findings are reproducible and have clinical effects. Furthermore, studies should focus on longitudinal designs that track changes in miRNA expressions from pre-surgery throughout various post-operative stages, relating these changes to clinical outcomes. Such efforts would help understand the variability of miRNA responses and could lead to miRNA-based interventions that optimize patient outcomes post-surgery, so miRNA can help predict the outcomes of the patients’ well-being after MBS. By addressing these challenges, future research can pave the way for integrating miRNA profiling into routine clinical practices, thereby enhancing the precision and effectiveness of treatments for patients undergoing MBS.

### Confounding and Bias

We recognize the critical necessity for a comprehensive analysis in this domain. Our review reveals a concerning gap: while 26.1% of the studies reviewed employed robust statistical methodologies to adjust for potential confounders, a majority (73.9%) did not.

This oversight underscores the urgent need to enhance the methodological rigor in future miRNA research to ensure the reliability and applicability of the findings in clinical settings.

To address this, future studies should prioritize the implementation of advanced statistical models across all miRNA research related to MBS. Incorporating such models will significantly improve the power to detect actual biological effects of miRNA changes, mitigating the influence of bias and confounding factors. This is crucial for transitioning from exploratory findings to actionable clinical interventions.

Moreover, our review highlights a critical knowledge gap regarding applying specific miRNA profiles in clinical practice. Which miRNA alterations predict or reflect specific clinical outcomes following different MBS procedures remains unclear. This ambiguity impedes the practical application of miRNA profiling in personalized patient care.

Future research should thus focus on longitudinal and cohort studies: These studies should correlate specific miRNA alterations with long-term clinical outcomes across different MBS procedures. This approach is essential to establish causal relationships and understand the role of miRNAs in patient recovery and complication rates post-MBS.

Standardization of miRNA profiling: developing standardized protocols for miRNA analysis in clinical settings will facilitate comparing results across studies and enhance the generalizability of findings and utilization of advanced analytics**.** Employing machine learning algorithms can further refine the predictive accuracy of miRNA profiles. These tools are adept at analyzing complex, large-scale datasets to unearth patterns that conventional statistical approaches may miss.

Incorporating these elements into future research will address the methodological deficiencies observed in the current literature and clarify which miRNAs are most pertinent for monitoring and enhancing patient outcomes in clinical practice. By committing to these research directions, we can better link miRNA molecular changes to practical clinical applications, paving the way for miRNAs to become integral components of personalized medicine in MBS.

In conclusion, the enhancement of methodological approaches and a focused investigation into the specific roles of miRNAs will be instrumental in transitioning from molecular observations to therapeutic and diagnostic utilities in clinical settings.

### Differences Between miRNA Notations

Another observation was the differences in the notation of specific miRNA with or without -3p or -5p. miRNAs are typically transcribed as primary miRNA transcripts (pri-miRNAs). The mature miRNA strand gets loaded onto the RNA-induced silencing complex (RISC) and performs gene silencing. A single pre-miRNA can give rise to two distinct mature miRNAs originating from the opposite arms of the pre-miRNA hairpin structure: one from the 5′ end (5p) and the other from the 3′ end (3p) [3, 34].

Historically, one of the arms (often the 5p) was typically more abundant and thus was primarily studied. However, advancements in sequencing technologies and further research have shown that both arms can give rise to functionally relevant miRNAs [3, 34].

The functional implications of the two mature miRNAs can vary. In some cases, both arms can target different sets of mRNAs and thus can have distinct biological roles. For instance, while miR-122-5p might target a set of genes involved in lipid metabolism, miR-122-3p could target a different set of genes or be present at too low levels to have a significant physiological impact [35, 36]. Simply mentioning “miR-122” can be ambiguous, as it does not specify which of the two mature products is being discussed. For clarity, accuracy, and comprehensive understanding, it is paramount that studies clearly distinguish between the 5p and 3p effects of miRNAs [37].

## Limitations

While our SR provides a comprehensive overview of the field, inherent limitations exist. Firstly, we could not capture all the miRNAs used in some of the included studies since this was not presented as what was used in their lab analysis, leading to a potential underestimation of the actual miRNAs studied. Lastly, while inclusive, the broad nature of our research question makes synthesizing information challenging and might prevent us from concluding specific associated medical problems or effects from MBS.

## Conclusion

Our systematic review shed light on the complex landscape of miRNA research. By identifying 507 distinct miRNAs and emphasizing the recurrent 16 miRNAs across studies, we spotlight potential foundational sets for future work. Instead of the standard approach of connecting medical problems to miRNAs, we traced outcomes from frequently cited miRNAs. This perspective, though intricate, unveils diverse outcomes tied to miRNAs. We observed inconsistent outcomes linked to the same miRNA, underscoring the need for clarity in miRNA-outcome relationships. The field grapples with diverse findings and methodological variations, pressing for standardized research methods. Addressing the unique 5p and 3p effects of miRNAs is crucial. Calls for consensus meetings to craft standardized miRNA sets might guide cohesive research, fostering enhanced comparisons and meta-analyses.

### Supplementary Information

ESM 1 (DOCX 30.8 KB)

ESM 2 (DOCX 15.2 KB)

ESM 3 (DOCX 35.8 KB)

ESM 4 (DOCX 27.0 KB)

## Data Availability

Data can be requested from the corresponding author.

## References

[1] Zhang Y, Proenca R, Maffei M, Barone M, Leopold L. Positional cloning of the mouse obese gene and its human homologue. 10.1038/372425a0 7984236

[2] Stefan M, Zhang W, Concepcion E, Yi Z, Tomer Y. DNA methylation profiles in type 1 diabetes twins point to strong epigenetic effects on etiology. J Autoimmun. 2014;50:33–7. 10.1016/j.jaut.2013.10.001 PMC3995844 24210274

[3] Bartel DP. MicroRNAs: target recognition and regulatory functions. Cell. 2009;136:215–33. 19167326 10.1016/j.cell.2009.01.002 PMC3794896

[4] Ortega FJ, Mercader JM, Catalán V, Moreno-Navarrete JM, Pueyo N, Sabater M, et al. Targeting the circulating MicroRNA signature of obesity. Clin Chem. 2013;59:781–92. 10.1373/clinchem.2012.195776 23396142

[5] Langi G, Szczerbinski L, Kretowski A. Meta-analysis of differential miRNA expression after bariatric surgery. JCM. 2019;8:1220. 31443156 10.3390/jcm8081220 PMC6723285

[6] Krol J, Loedige I, Filipowicz W. The widespread regulation of microRNA biogenesis, function and decay. Nat Rev Genet. 2010;11:597–610. 20661255 10.1038/nrg2843

[7] Moher D, Liberati a, Tetzlaff J, Altman DG, Grp P. Preferred reporting items for systematic reviews and meta-analyses: the PRISMA statement (Reprinted from Annals of Internal Medicine). Phys Ther. 2009;89:873–80. 19723669

[8] Wells GA, Shea B, O’Connell D, Peterson J, Welch V, Losos M, et al. The Newcastle-Ottawa Scale (NOS) for assessing the quality of nonrandomised studies in meta-analyses. https://www.ohri.ca/programs/clinical_epidemiology/oxford.asp. Accessed 17 Apr 2024.

[9] Yeh J, Chen C, Liu K, Peng C, Lin T, Chang Y, et al. Serum microRNA panels predict bariatric surgery outcomes. Obesity. 2022;30:389–99. 10.1002/oby.23330 35088552

[10] Hany M, Demerdash H, Ahmed AE, Agayby AS, Ghaballa M, Ibrahim M, et al. MicroRNA profiling and the effect on metabolic biomarkers and weight loss after laparoscopic sleeve gastrectomy: a prospective cohort study. J Bariatr Surg. 2022;1:88.

[11] Eikelis N, Dixon JB, Lambert EA, Hanin G, Tzur Y, Greenberg DS, et al. MicroRNA-132 may be associated with blood pressure and liver steatosis—preliminary observations in obese individuals. J Hum Hypertens. 2022;36:911–6. 10.1038/s41371-021-00597-2 34453104

[12] Breininger SP, Sabater L, Malcomson FC, Afshar S, Mann J, Mathers JC. Obesity and Roux-en-Y gastric bypass drive changes in miR-31 and miR-215 expression in the human rectal mucosa. Int J Obes. 2022;46:333–41. 10.1038/s41366-021-01005-y PMC8794786 34716428

[13] Sangiao-Alvarellos S, Theofilatos K, Barwari T, Gutmann C, Takov K, Singh B, et al. Metabolic recovery after weight loss surgery is reflected in serum microRNAs. BMJ Open Diab Res Care. 2020;8:e001441. 10.1136/bmjdrc-2020-001441 PMC7594349 33115818

[14] Doyon L, Das S, Sullivan T, Rieger-Christ K, Sherman J, Roque S, et al. Can genetics help predict efficacy of bariatric surgery? an analysis of microRNA profiles. Surg Obes Relat Dis. 2020;16:1802–7. 10.1016/j.soard.2020.06.024 32737014

[15] Cereijo R, Taxerås SD, Piquer-Garcia I, Pellitero S, Martínez E, Tarascó J, et al. Elevated levels of circulating miR-92a are associated with impaired glucose homeostasis in patients with obesity and correlate with metabolic status after bariatric Surgery. Obes Surg. 2020;30:174–9. 10.1007/s11695-019-04104-y 31346930

[16] Bae Y, Kim Y, Lee H, Kim H, Jeon JS, Noh H, et al. Bariatric surgery alters microRNA content of circulating exosomes in patients with obesity. Obesity. 2019;27:264–71. 10.1002/oby.22379 30624857

[17] Atkin SL, Ramachandran V, Yousri NA, Benurwar M, Simper SC, McKinlay R, et al. Changes in blood microRNA expression and early metabolic responsiveness 21 days following bariatric surgery. Front Endocrinol. 2019;9:773. 10.3389/fendo.2018.00773 PMC6338028 30687230

[18] Wang Y, Wang D-S, Cheng Y-S, Jia B-L, Yu G, Yin X-Q, et al. Expression of MicroRNA-448 and SIRT1 and prognosis of obese type 2 diabetic mellitus patients after laparoscopic bariatric surgery. Cell Physiol Biochem. 2018;45:935–50. 10.1159/000487287 29428938

[19] Liao C-H, Wang C-Y, Liu K-H, Liu Y-Y, Wen M-S, Yeh T-S. MiR-122 marks the differences between subcutaneous and visceral adipose tissues and associates with the outcome of bariatric surgery. Obes Res Clin Pract. 2018;12:570–7. 10.1016/j.orcp.2018.06.005 29960868

[20] Hohensinner PJ, Kaun C, Ebenbauer B, Hackl M, Demyanets S, Richter D, et al. Reduction of premature aging markers after gastric bypass surgery in morbidly obese patients. Obes Surg. 2018;28:2804–10. 10.1007/s11695-018-3247-3 PMC6132736 29693219

[21] Alkandari A, Ashrafian H, Sathyapalan T, Sedman P, Darzi A, Holmes E, et al. Improved physiology and metabolic flux after Roux-en-Y gastric bypass is associated with temporal changes in the circulating microRNAome: a longitudinal study in humans. BMC Obes. 2018;5:20. 10.1186/s40608-018-0199-z PMC5984421 29881628

[22] Nunez Lopez YO, Coen PM, Goodpaster BH, Seyhan AA. Gastric bypass surgery with exercise alters plasma microRNAs that predict improvements in cardiometabolic risk. Int J Obes. 2017;41:1121–30. 10.1038/ijo.2017.84 PMC5576445 28344345

[23] Nardelli C, Iaffaldano L, Pilone V, Labruna G, Ferrigno M, Carlomagno N, et al. Changes in the MicroRNA profile observed in the subcutaneous adipose tissue of obese patients after laparoscopic adjustable gastric banding. J Obes. 2017;2017:1–6. 10.1155/2017/6754734 PMC5366784 28386478

[24] Mysore R, Ortega FJ, Latorre J, Ahonen M, Savolainen-Peltonen H, Fischer-Posovszky P, et al. MicroRNA-221-3p regulates angiopoietin-like 8 (ANGPTL8) expression in adipocytes. J Clin Endocrinol Metabol. 2017;102:4001–12. 10.1210/jc.2017-00453 28938482

[25] Hubal MJ, Nadler EP, Ferrante SC, Barberio MD, Suh J-H, Wang J, et al. Circulating adipocyte-derived exosomal MicroRNAs associated with decreased insulin resistance after gastric bypass: gastric bypass alters exosomal MicroRNAs. Obesity. 2017;25:102–10. 10.1002/oby.21709 PMC5182153 27883272

[26] Blum A, Yehuda H, Geron N, Meerson A. Elevated levels of miR-122 in serum may contribute to improved endothelial function and lower oncologic risk following bariatric surgery. 2017. 29103239

[27] Ortega FJ, Moreno M, Mercader JM, Moreno-Navarrete JM, Fuentes-Batllevell N, Sabater M, et al. Inflammation triggers specific microRNA profiles in human adipocytes and macrophages and in their supernatants. Clin Epigenet. 2015;7:49. 10.1186/s13148-015-0083-3 PMC4413548 25926893

[28] Ortega FJ, Mercader JM, Moreno-Navarrete JM, Nonell L, Puigdecanet E, Rodriquez-Hermosa JI, et al. Surgery-induced weight loss is associated with the downregulation of genes targeted by MicroRNAs in adipose tissue. J Clin Endocrinol Metabol. 2015;100:E1467–76. 10.1210/jc.2015-2357 26252355

[29] Lirun K, Sewe M, Yong W. A pilot study: the effect of Roux-en-Y gastric bypass on the serum MicroRNAs of the type 2 diabetes patient. Obes Surg. 2015;25:2386–92. 10.1007/s11695-015-1711-x 26138690

[30] Hulsmans M, Sinnaeve P, Van Der Schueren B, Mathieu C, Janssens S, Holvoet P. Decreased miR-181a expression in monocytes of obese patients is associated with the occurrence of metabolic syndrome and coronary artery disease. J Clin Endocrinol Metabol. 2012;97:E1213–8. 10.1210/jc.2012-1008 22535975

[31] Macartney‐Coxson D, Danielson K, Clapham J, Benton MC, Johnstone A, Jones A, et al. MicroRNA profiling in adipose before and after weight loss highlights the role of miR‐223‐3p and the NLRP3 inflammasome. Obesity. 2020;28:570–80. 10.1002/oby.22722 PMC7046053 32090515

[32] Kuryłowicz A, Wicik Z, Owczarz M, Jonas M, Kotlarek M, Świerniak M, et al. NGS reveals molecular pathways affected by obesity and weight loss-related changes in miRNA levels in adipose tissue. IJMS. 2017;19:66. 10.3390/ijms19010066 PMC5796016 29280944

[33] Catanzaro G, Filardi T, Sabato C, Vacca A, Migliaccio S, Morano S, et al. Tissue and circulating microRNAs as biomarkers of response to obesity treatment strategies. J Endocrinol Invest. 2021;44:1159–74. 10.1007/s40618-020-01453-9 PMC8124039 33111214

[34] Ambros V. The functions of animal microRNAs. Nature. 2004;431:350–5. 10.1038/nature02871 15372042

[35] Griffiths-Jones S. MiRBase: microRNA sequences, targets and gene nomenclature. Nucleic Acids Res. 2006;34:D140–4. 10.1093/nar/gkj112 PMC1347474 16381832

[36] Yang J-S, Lai EC. Alternative miRNA biogenesis pathways and the interpretation of core miRNA pathway mutants. Molecular Cell. 2011;43:892–903. 10.1016/j.molcel.2011.07.024 PMC3176435 21925378

[37] Chiang HR, Schoenfeld LW, Ruby JG, Auyeung VC, Spies N, Baek D, et al. Mammalian microRNAs: experimental evaluation of novel and previously annotated genes. Genes Dev. 2010;24:992–1009. 10.1101/gad.1884710 PMC2867214 20413612

