# Supplementary material for: Expression and Relations of Unique miRNAs Investigated in Metabolic Bariatric Surgery: A Systematic Review
Source: Obes Surg. 2024 Jun 25;34(8):3038–57. doi: 10.1007/s11695-024-07302-5 (PMC11289332; doi:10.1007/s11695-024-07302-5)
Supplement: Supplementary file 2 — (DOCX 15.2 KB) [file 11695_2024_7302_MOESM2_ESM.docx]

**Appendix 2: Search strategy databses:**

**PubMed**

(miRNA* OR microRNA* OR "messenger RNA*") AND ("bariatric surgery" OR "metabolic surgery" OR RYGB OR "Roux-en-Y gastric bypass" OR LSG OR "laparoscopic sleeve gastrectomy" OR OAGB OR "one anastomosis gastric bypass")

***Notes****: Truncation (*) is used to capture various endings. Quotation marks are used to ensure that phrases are searched exactly as intended.*

**EMBASE:**

('miRNA'/exp OR 'miRNA'* OR 'microRNA'/exp OR 'microRNA'* OR 'messenger RNA'/exp OR 'messenger RNA'*) AND ('bariatric surgery'/exp OR 'metabolic surgery'/exp OR 'RYGB'/exp OR 'Roux-en-Y gastric bypass'/exp OR 'LSG'/exp OR 'laparoscopic sleeve gastrectomy'/exp OR 'OAGB'/exp OR 'one anastomosis gastric bypass'/exp)

***Notes****: Using* ***/exp*** *expands the search to include all relevant Emtree terms automatically. This ensures that the search is as comprehensive as possible, covering indexed terms related to both miRNA and different surgical procedures.*

**Cochrane Central Register of Controlled Trials (CENTRAL)**:

(miRNA* OR microRNA* OR "messenger RNA*") in Trials AND ("bariatric surgery" OR "metabolic surgery" OR RYGB OR "Roux-en-Y gastric bypass" OR LSG OR "laparoscopic sleeve gastrectomy" OR OAGB OR "one anastomosis gastric bypass" in Trials)
