# Supplementary material for: Expression and Relations of Unique miRNAs Investigated in Metabolic Bariatric Surgery: A Systematic Review
Source: Obes Surg. 2024 Jun 25;34(8):3038–57. doi: 10.1007/s11695-024-07302-5 (PMC11289332; doi:10.1007/s11695-024-07302-5)
Supplement: Supplementary file 3 — (DOCX 35.8 KB) [file 11695_2024_7302_MOESM3_ESM.docx]

**Appendix 3: All presented miRNA in number of studies**

| **RNA** | **Number of Studies** |
| --- | --- |
| 1-3p | 1 |
| 100 | 1 |
| 100-3p | 1 |
| 100-5 p | 2 |
| 101-3p | 2 |
| 101-5p | 2 |
| 103-3p | 1 |
| 103a | 1 |
| 106a | 2 |
| 106a-5p | 2 |
| 106b-3p | 2 |
| 106b-5p | 4 |
| 107 | 3 |
| 10a | 1 |
| 10a-5p | 1 |
| 10b-5p | 2 |
| 122 | 3 |
| 122-5p | 7 |
| 1224-5p | 1 |
| 1227-3p | 1 |
| 124 | 1 |
| 1246 | 3 |
| 1247-3p | 1 |
| 1247-5p | 1 |
| 1248 | 1 |
| 125-1-3p | 1 |
| 1253 | 1 |
| 1256-5p | 1 |
| 125a-5p | 3 |
| 125b | 2 |
| 125b-5p | 3 |
| 126 | 1 |
| 126-3 p | 1 |
| 126-3p | 2 |
| 126-5p | 3 |
| 1260a | 1 |
| 1260b | 2 |
| 1262 | 1 |
| 1268a | 1 |
| 127-3p | 1 |
| 1271-5p | 1 |
| 1273_d | 1 |
| 1273a | 2 |
| 1273c | 2 |
| 1273e | 2 |
| 1273f | 1 |
| 1273g-3p | 1 |
| 128 | 3 |
| 128-3p | 1 |
| 1281 | 1 |
| 1285-3p | 3 |
| 1290 | 1 |
| 1295 | 1 |
| 130 | 1 |
| 1301 | 1 |
| 1307-5p | 1 |
| 130a | 1 |
| 130a-3p | 1 |
| 130b | 2 |
| 130b | 1 |
| 130b-3p | 2 |
| 132 | 2 |
| 132-3p | 1 |
| 132-5p | 1 |
| 133a | 1 |
| 133a-3p | 1 |
| 133b | 1 |
| 135b-5p | 1 |
| 136-3p | 1 |
| 136-5p | 1 |
| 138-5p | 1 |
| 140-5p | 4 |
| 141-3p | 1 |
| 142-3p | 3 |
| 142-5p | 1 |
| 143-3p | 2 |
| 143-5p | 2 |
| 144-3p | 2 |
| 144-5p | 1 |
| 145 | 2 |
| 145-3p | 1 |
| 145-5p | 2 |
| 146 | 1 |
| 1469 | 1 |
| 146a | 2 |
| 146a-3p | 1 |
| 146a-5p | 1 |
| 146b | 1 |
| 146b-3p | 1 |
| 146b-5p | 2 |
| 147b | 1 |
| 148a-3p | 3 |
| 148a-5p | 1 |
| 148b-3p | 3 |
| 149 | 1 |
| 149-5p | 1 |
| 150-5p | 3 |
| 151-3p | 1 |
| 151-5p | 1 |
| 151a-5p | 1 |
| 152 | 1 |
| 154-5p | 1 |
| 155 | 2 |
| 155-5p | 1 |
| 15a | 1 |
| 15a-3p | 1 |
| 15a-5p | 3 |
| 15b | 1 |
| 15b-3p | 2 |
| 15b-5p | 3 |
| 16 | 2 |
| 16-2-3p | 2 |
| 16-5p | 3 |
| 17 | 2 |
| 17-3p | 1 |
| 17-5p | 1 |
| 18 | 1 |
| 181_d | 1 |
| 181a | 2 |
| 181a-3p | 1 |
| 181a-5p | 1 |
| 181b | 1 |
| 182-5p | 2 |
| 1825 | 1 |
| 183-5p | 4 |
| 184 | 1 |
| 185-5p | 2 |
| 186-5p | 2 |
| 18a-3p | 1 |
| 18a-5p | 2 |
| 18b | 1 |
| 18b-5p | 1 |
| 19 | 1 |
| 190 | 1 |
| 190a | 1 |
| 191 | 1 |
| 191-5p | 3 |
| 192-5p | 3 |
| 193a-3p | 2 |
| 193a-5p | 2 |
| 193b | 1 |
| 193b-3p | 2 |
| 193b-5p | 2 |
| 194-5p | 5 |
| 195 | 1 |
| 196a-5p | 3 |
| 196b-5p | 1 |
| 1973 | 1 |
| 199a-3p | 2 |
| 199a-5p | 1 |
| 199b-5p | 4 |
| 19a | 1 |
| 19a-3p | 1 |
| 19b | 2 |
| 19b-3p | 3 |
| 200a | 1 |
| 200a-3p | 2 |
| 200a-5p | 1 |
| 200b-3p | 2 |
| 200b-5p | 1 |
| 200c | 1 |
| 200c-3p | 2 |
| 203a | 1 |
| 203a-3p | 1 |
| 204 | 1 |
| 204-5p | 2 |
| 205 | 1 |
| 205-5p | 2 |
| 206 | 1 |
| 208a-3p | 2 |
| 20a | 2 |
| 20a-5p | 1 |
| 20b-5p | 4 |
| 21 | 3 |
| 21-5p | 5 |
| 210 | 1 |
| 210-3p | 2 |
| 210-5p | 1 |
| 2110 | 1 |
| 212 | 2 |
| 214 | 1 |
| 214-3p | 1 |
| 214-5p | 1 |
| 215 | 1 |
| 215-3p | 1 |
| 215-5p | 1 |
| 218-5p | 1 |
| 219a-5p | 1 |
| 22 | 2 |
| 22-3p | 2 |
| 22-5p | 1 |
| 221 | 5 |
| 221-3p | 3 |
| 222 | 2 |
| 222-3p | 1 |
| 222-5p | 1 |
| 223 | 1 |
| 223-3p | 6 |
| 223-5p | 2 |
| 224 | 1 |
| 224-5p | 1 |
| 2277-5p | 1 |
| 2355-5p | 1 |
| 23a | 1 |
| 23a-5p | 3 |
| 23b | 1 |
| 24 | 2 |
| 24-1-5p | 1 |
| 24–2-5p | 1 |
| 25 | 2 |
| 25-3p | 3 |
| 25-5p | 1 |
| 26a | 1 |
| 26a-5p | 1 |
| 26b-3p | 1 |
| 27 | 1 |
| 27 a-3 p | 1 |
| 27a | 1 |
| 27a-3p | 2 |
| 27a-5p | 2 |
| 27b | 1 |
| 27b-3p | 3 |
| 28-3p | 2 |
| 28-5p | 1 |
| 2861 | 1 |
| 296-5p | 1 |
| 299-3p | 1 |
| 299-5p | 1 |
| 29a-3p | 3 |
| 29a-5p | 1 |
| 29b-3p | 3 |
| 29c | 1 |
| 29c-3p | 3 |
| 29c-5p | 1 |
| 30 | 1 |
| 30 c-5 p | 1 |
| 30_d | 1 |
| 301a-3p | 1 |
| 301b | 1 |
| 30a-3p | 1 |
| 30a-5p | 2 |
| 30b-5p | 2 |
| 30c-2-3p | 1 |
| 30e | 1 |
| 30e-3p | 2 |
| 30e-5p | 1 |
| 31 | 1 |
| 31-3p | 1 |
| 31-5p | 3 |
| 3127-5p | 1 |
| 3135b | 1 |
| 3150b-3p | 1 |
| 3178 | 1 |
| 3196 | 1 |
| 32-5p | 2 |
| 3200-3p | 1 |
| 320a | 5 |
| 320b | 3 |
| 320c | 2 |
| 323-3p | 1 |
| 324-5p | 1 |
| 328 | 1 |
| 328-3p | 1 |
| 330-3p | 1 |
| 331-5p | 1 |
| 335-5p | 2 |
| 337-3p | 1 |
| 338-3p | 2 |
| 339 –3p | 1 |
| 339-3p | 1 |
| 339-5p | 1 |
| 33a-3p | 1 |
| 33a-5p | 2 |
| 33b-5p | 1 |
| 340-3p | 1 |
| 340-5p | 1 |
| 342-3p | 3 |
| 342-5p | 2 |
| 345 | 2 |
| 34a | 1 |
| 34a-5p | 1 |
| 3607-3p | 1 |
| 361-3p | 1 |
| 3615 | 1 |
| 362-3p | 2 |
| 3622a-3p | 1 |
| 363-3p | 2 |
| 3648 | 1 |
| 365 | 1 |
| 3656 | 1 |
| 365a-3p | 2 |
| 365b-3p | 1 |
| 3665 | 1 |
| 3688-3p | 2 |
| 369-5p | 1 |
| 3690 | 1 |
| 370 | 1 |
| 374 | 1 |
| 374b-5p | 1 |
| 375 | 1 |
| 375-3p | 2 |
| 376a-3p | 1 |
| 376a-5p | 1 |
| 376c | 1 |
| 376c-3p | 1 |
| 378 | 3 |
| 378a-3p | 2 |
| 378c | 1 |
| 378g | 1 |
| 381-3p | 1 |
| 382 | 1 |
| 382-3p | 1 |
| 3909 | 1 |
| 3926 | 1 |
| 3960 | 2 |
| 411 | 1 |
| 421 | 2 |
| 422a | 2 |
| 423 | 1 |
| 423-3p | 3 |
| 423-5p | 2 |
| 424-3p | 2 |
| 424-5p | 4 |
| 425 | 2 |
| 425-5p | 1 |
| 4271 | 1 |
| 4284 | 2 |
| 4286 | 1 |
| 429 | 2 |
| 432-5p | 1 |
| 4429 | 1 |
| 4446-3p | 1 |
| 4449 | 1 |
| 4459 | 1 |
| 4461 | 1 |
| 4473 | 1 |
| 448 | 1 |
| 4485 | 1 |
| 4485-5p | 1 |
| 4487 | 1 |
| 4497 | 2 |
| 4508 | 1 |
| 450a-5p | 2 |
| 450b-5p | 1 |
| 451 | 2 |
| 4516 | 1 |
| 451a | 1 |
| 452-5p | 1 |
| 4525 | 1 |
| 4530 | 1 |
| 455-3p | 1 |
| 455-5p | 1 |
| 4662a-5p | 1 |
| 4664-5p | 1 |
| 4691-5p | 1 |
| 4709-5p | 1 |
| 4716-3p | 1 |
| 4723-5p | 1 |
| 4728-3p | 1 |
| 4732-3p | 1 |
| 4747-5p | 1 |
| 4749-3p | 1 |
| 4763-3p | 1 |
| 4782-5p | 1 |
| 4787-5p | 1 |
| 4792 | 1 |
| 483-3p | 1 |
| 483-5p | 1 |
| 483–5p | 1 |
| 484 | 1 |
| 485-3p | 1 |
| 486 | 1 |
| 486-3p | 3 |
| 486-5p | 4 |
| 487a | 1 |
| 487b | 1 |
| 489 | 1 |
| 493-3p | 1 |
| 494 | 1 |
| 495-3p | 1 |
| 496 | 1 |
| 497-5p | 3 |
| 499a-Sp | 1 |
| 500a-3p | 1 |
| 501–3p | 2 |
| 502-3p | 3 |
| 503-5p | 1 |
| 505-3p | 1 |
| 5096 | 1 |
| 516b-5p | 1 |
| 518b | 1 |
| 519 | 1 |
| 520a-3p | 1 |
| 520c-3p | 1 |
| 520g | 1 |
| 532-3p | 3 |
| 532-5p | 3 |
| 539-3p | 1 |
| 539-5p | 1 |
| 543 | 1 |
| 548ay-5p | 1 |
| 548c-5p | 1 |
| 548d-5p | 1 |
| 548f-5p | 1 |
| 548h-5p | 1 |
| 550a-3p | 1 |
| 551b-3p | 1 |
| 552-3p | 1 |
| 552-5p | 1 |
| 561-5p | 1 |
| 566 | 1 |
| 5683 | 1 |
| 572 | 1 |
| 574-3p | 2 |
| 575 | 1 |
| 576-5p | 1 |
| 584-5p | 1 |
| 589-5p | 1 |
| 590-5 p | 1 |
| 590-5p | 2 |
| 6087 | 1 |
| 6126 | 1 |
| 615-3p | 1 |
| 615-5p | 1 |
| 619-5p | 1 |
| 625 | 1 |
| 625-3p | 1 |
| 625-5p | 1 |
| 628-5p | 1 |
| 629-5p | 2 |
| 636 | 1 |
| 638 | 1 |
| 642b-5p | 1 |
| 652 | 1 |
| 652-3p | 3 |
| 655 | 1 |
| 655-5p | 1 |
| 660-5p | 1 |
| 663 a | 1 |
| 664a-3p | 1 |
| 671-3p | 3 |
| 671-5p | 1 |
| 6716-3p | 1 |
| 6739-5p | 1 |
| 6751-3p | 1 |
| 7_d | 1 |
| 7-5p | 4 |
| 708 | 1 |
| 7110-5p | 1 |
| 744 | 1 |
| 744-5p | 1 |
| 758 | 1 |
| 762 | 1 |
| 7641-2 | 1 |
| 766 | 1 |
| 766-3p | 1 |
| 7706 | 1 |
| 7847-3p | 1 |
| 7851-3p | 1 |
| 7a-3p | 1 |
| 7d-3p | 2 |
| 7e-3p | 1 |
| 7f-1-3p | 1 |
| 7i-5p | 1 |
| 8485 | 1 |
| 874 | 1 |
| 874-3p | 2 |
| 877-5p | 1 |
| 885-5p | 2 |
| 887 | 1 |
| 887-3p | 1 |
| 891a | 1 |
| 9-5p | 3 |
| 92a | 4 |
| 92a-3p | 3 |
| 92b-3p | 3 |
| 93 | 2 |
| 93-3p | 1 |
| 93-5p | 4 |
| 941 | 1 |
| 95 | 2 |
| 96-5p | 1 |
| 99a-5p | 2 |
| 99b | 1 |
| 99b-5p | 3 |
| IL-6 mRNA | 1 |
| let-7 | 1 |
| let-7a | 3 |
| let-7a-5p | 1 |
| let-7b | 1 |
| let-7b-5p | 1 |
| let-7c | 1 |
| let-7d | 1 |
| let-7d-3p | 1 |
| let-7d-5p | 1 |
| let-7e | 1 |
| let-7f | 1 |
| let-7f-5p | 1 |
| let-7i | 1 |
| let-7i-3p | 1 |
| let-7i-5p | 2 |
| let7b | 1 |
| let7f-1-3p | 1 |
| NLRP3 | 1 |
| RNU48 | 1 |
| sirt1 | 1 |
| TNFα mRNA | 1 |
