# Supplementary material for: Expression and Relations of Unique miRNAs Investigated in Metabolic Bariatric Surgery: A Systematic Review
Source: Obes Surg. 2024 Jun 25;34(8):3038–57. doi: 10.1007/s11695-024-07302-5 (PMC11289332; doi:10.1007/s11695-024-07302-5)
Supplement: Supplementary file 4 — (DOCX 27.0 KB) [file 11695_2024_7302_MOESM4_ESM.docx]

**Appendix 4: Total amount of studies with miRNA with down and/or up-regulated effects after bariatric metabolic surgery.**

| **RNA** | **down** | **up** |
| --- | --- | --- |
| 100 | 0 | 1 |
| 103-3p | 0 | 1 |
| 106b-5p | 2 | 1 |
| 107 | 1 | 0 |
| 10a | 0 | 1 |
| 10b-5p | 0 | 1 |
| 122 | 1 | 2 |
| 122-5p | 2 | 1 |
| 1224-5p | 1 | 0 |
| 1227-3p | 0 | 1 |
| 1246 | 1 | 1 |
| 1256-5p | 1 | 0 |
| 125b | 0 | 1 |
| 125b-5p | 2 | 1 |
| 126-3p | 0 | 1 |
| 128 | 1 | 1 |
| 1290 | 0 | 1 |
| 1295 | 0 | 1 |
| 1301 | 1 | 0 |
| 130a | 0 | 1 |
| 130b | 0 | 2 |
| 130b | 1 | 0 |
| 130b-3p | 1 | 0 |
| 132 | 1 | 0 |
| 135b-5p | 1 | 0 |
| 138-5p | 1 | 0 |
| 140-5p | 1 | 0 |
| 142-3p | 1 | 0 |
| 144-3p | 0 | 1 |
| 145 | 0 | 1 |
| 146a-3p | 0 | 1 |
| 146b-3p | 0 | 1 |
| 146b-5p | 0 | 2 |
| 148a-3p | 1 | 0 |
| 148b-3p | 1 | 0 |
| 149-5p | 1 | 0 |
| 155 | 1 | 0 |
| 155-5p | 0 | 1 |
| 15a-5p | 2 | 1 |
| 16 | 1 | 0 |
| 16-2-3p | 1 | 0 |
| 16-5p | 1 | 0 |
| 181_d | 1 | 0 |
| 181a | 1 | 0 |
| 181a-5p | 0 | 1 |
| 181b | 1 | 0 |
| 183-5p | 1 | 0 |
| 184 | 0 | 1 |
| 18b | 1 | 0 |
| 190 | 0 | 1 |
| 192-5p | 4 | 0 |
| 193a-3p | 1 | 0 |
| 193a-5p | 1 | 0 |
| 193b-3p | 1 | 0 |
| 193b-5p | 0 | 1 |
| 194-5p | 3 | 0 |
| 195 | 0 | 1 |
| 196a-5p | 1 | 0 |
| 1973 | 0 | 1 |
| 199a-5p | 0 | 1 |
| 199b-5p | 0 | 1 |
| 200a | 1 | 0 |
| 200a-3p | 0 | 1 |
| 200b-3p | 0 | 1 |
| 200c | 1 | 0 |
| 200c-3p | 1 | 0 |
| 204 | 0 | 1 |
| 204-5p | 0 | 1 |
| 205 | 1 | 0 |
| 205-5p | 1 | 0 |
| 206 | 1 | 0 |
| 208a-3p | 2 | 0 |
| 20b-5p | 1 | 0 |
| 21 | 1 | 2 |
| 21-5p | 2 | 0 |
| 212 | 0 | 1 |
| 215-3p | 1 | 0 |
| 219a-5p | 0 | 1 |
| 22 | 3 | 0 |
| 22-5p | 2 | 0 |
| 221 | 1 | 2 |
| 221-3p | 2 | 0 |
| 222 | 1 | 0 |
| 222-5p | 1 | 0 |
| 223-3p | 1 | 1 |
| 223-5p | 1 | 0 |
| 224 | 0 | 1 |
| 224-5p | 1 | 0 |
| 2355-5p | 1 | 0 |
| 23a-5p | 2 | 0 |
| 24–2-5p | 1 | 0 |
| 27a-3p | 0 | 1 |
| 27a-5p | 2 | 0 |
| 27b | 0 | 1 |
| 28-3p | 1 | 0 |
| 296-5p | 0 | 1 |
| 299-5p | 0 | 1 |
| 29a-5p | 1 | 0 |
| 29b-3p | 1 | 0 |
| 29c | 0 | 1 |
| 29c-3p | 2 | 0 |
| 301a-3p | 0 | 2 |
| 30_d | 0 | 1 |
| 30e | 1 | 0 |
| 30e-3p | 1 | 0 |
| 31-5p | 2 | 1 |
| 3178 | 1 | 0 |
| 32-5p | 2 | 0 |
| 320a | 4 | 0 |
| 320b | 1 | 0 |
| 323-3p | 0 | 1 |
| 328 | 0 | 1 |
| 328-3p | 0 | 1 |
| 331-5p | 1 | 0 |
| 338-3p | 1 | 0 |
| 339 –3p | 1 | 0 |
| 339-3p | 1 | 0 |
| 339-5p | 0 | 1 |
| 33a-5p | 0 | 1 |
| 342-5p | 1 | 0 |
| 345 | 1 | 0 |
| 34a | 0 | 1 |
| 34a-5p | 1 | 0 |
| 362-3p | 0 | 1 |
| 3622a-3p | 0 | 1 |
| 363-3p | 2 | 0 |
| 365 | 0 | 1 |
| 365a-3p | 1 | 0 |
| 3690 | 1 | 0 |
| 370 | 1 | 0 |
| 374b-5p | 0 | 3 |
| 375 | 1 | 0 |
| 378 | 0 | 1 |
| 378a-3p | 3 | 0 |
| 378c | 0 | 1 |
| 378g | 0 | 1 |
| 382 | 1 | 0 |
| 3926 | 1 | 0 |
| 421 | 1 | 0 |
| 423 | 1 | 0 |
| 423-3p | 2 | 0 |
| 423-5p | 0 | 1 |
| 424-3p | 0 | 1 |
| 424-5p | 1 | 1 |
| 4286 | 1 | 0 |
| 429 | 1 | 1 |
| 4449 | 0 | 1 |
| 448 | 1 | 0 |
| 450b-5p | 0 | 1 |
| 451 | 1 | 0 |
| 4525 | 1 | 0 |
| 4664-5p | 1 | 0 |
| 4691-5p | 0 | 1 |
| 4716-3p | 1 | 0 |
| 4723-5p | 1 | 0 |
| 4728-3p | 0 | 1 |
| 4747-5p | 1 | 0 |
| 4749-3p | 0 | 1 |
| 4782-5p | 1 | 0 |
| 483-5p | 1 | 0 |
| 483–5p | 1 | 0 |
| 486 | 1 | 0 |
| 486-5p | 1 | 0 |
| 487a | 1 | 0 |
| 493-3p | 0 | 1 |
| 494 | 0 | 1 |
| 496 | 1 | 0 |
| 497-5p | 1 | 0 |
| 499a-Sp | 0 | 1 |
| 501–3p | 1 | 0 |
| 502-3p | 2 | 0 |
| 503-5p | 0 | 1 |
| 505-3p | 1 | 0 |
| 516b-5p | 0 | 1 |
| 519 | 0 | 1 |
| 520a-3p | 1 | 0 |
| 520g | 0 | 1 |
| 532-3p | 1 | 1 |
| 532-5p | 1 | 0 |
| 539-5p | 0 | 1 |
| 548c-5p | 1 | 0 |
| 574-3p | 0 | 1 |
| 575 | 0 | 1 |
| 590-5p | 1 | 0 |
| 6126 | 0 | 1 |
| 615-3p | 1 | 0 |
| 615-5p | 1 | 0 |
| 629-5p | 2 | 0 |
| 652 | 0 | 1 |
| 655 | 0 | 1 |
| 660-5p | 2 | 0 |
| 671-3p | 0 | 1 |
| 7-5p | 0 | 1 |
| 708 | 0 | 1 |
| 744 | 0 | 1 |
| 758 | 0 | 1 |
| 762 | 1 | 0 |
| 7641-2 | 0 | 1 |
| 766-3p | 1 | 0 |
| 7d-3p | 1 | 0 |
| 7i-5p | 1 | 0 |
| 874-3p | 0 | 1 |
| 885-5p | 1 | 0 |
| 9-5p | 1 | 0 |
| 92a | 1 | 1 |
| 92a-3p | 2 | 0 |
| 93-3p | 1 | 0 |
| 93-5p | 1 | 0 |
| 95 | 0 | 1 |
| 96-5p | 1 | 0 |
| 99a-5p | 2 | 0 |
| 99b | 0 | 1 |
| NLRP3 | 1 | 0 |
| let-7a | 0 | 1 |
| let-7b-5p | 1 | 0 |
| let-7d | 0 | 1 |
| let-7d-3p | 1 | 0 |
| let-7e | 0 | 1 |
| let-7f | 0 | 1 |
| let-7i-5p | 1 | 0 |
| sirt1 | 0 | 1 |
